# Abundance of Common Aerobic Anoxygenic Phototrophic Bacteria in a Coastal Aquaculture Area

**DOI:** 10.3389/fmicb.2016.01996

**Published:** 2016-12-15

**Authors:** Yuki Sato-Takabe, Hironori Nakao, Takafumi Kataoka, Taichi Yokokawa, Koji Hamasaki, Kohei Ohta, Satoru Suzuki

**Affiliations:** ^1^Center for Marine Environmental Studies, Ehime UniversityMatsuyama, Japan; ^2^Atmosphere and Ocean Research Institute, The University of TokyoKashiwa, Japan; ^3^South Ehime Fisheries Research Center, Ehime UniversityAinan, Japan

**Keywords:** aerobic anoxygenic phototrophic bacteria, biomass, phylogeny, aquaculture, Uwa Sea

## Abstract

Aerobic anoxygenic phototrophic bacteria (AAnPB) rely on not only heterotrophic but also phototrophic energy gain. AAnPB are known to have high abundance in oligotrophic waters and are the major portion of the bacterial carbon stock in the environment. In a yearlong study in an aquaculture area in the Uwa Sea, Japan, AAnPB, accounted for 4.7 to 24% of the total bacteria by count. Since the cell volume of AAnPB is 2.23 ± 0.674 times larger than the mean for total bacteria, AAnPB biomass is estimated to account for 10–53% of the total bacterial assemblage. By examining *pufM* gene sequence, a common phylogenetic AAnPB species was found in all sampling sites through the year. The common species and other season-specific species were phylogenetically close to unculturable clones recorded in the Sargasso Sea and Pacific Ocean. The present study suggests that the common species may be a cosmopolitan species with worldwide distribution that is abundant not only in the oligotrophic open ocean but also in eutrophic aquaculture areas.

## Introduction

Aerobic anoxygenic phototrophic bacteria (AAnPB) are heterotrophic bacteria but have bacteriochlorophyll *a* (BChl *a*) and aerobically produce ATP through the photosynthetic pathway. AAnPB are known to be widely distributed in the ocean (Kolber et al., 2001; Lami et al., 2007; Cottrell and Kirchman, 2009; Lamy et al., 2011) with the highest abundances of 24% of total bacterial community reported in oligotrophic conditions in the South Pacific Ocean (Lami et al., 2007). This suggests that AAnPB could play an important role as a large part of the bacterial carbon stock in oligotrophic open oceans. Since organic carbon inputs at coastal aquaculture sites are expected to be high, the contribution of AAnPB at those sites could also be high as a carbon reservoir. However, little is known about AAnPB dynamics in the eutrophic coastal sea with respect to aquaculture activity.

Aerobic anoxygenic phototrophic bacteria cells are generally bigger than other heterotrophic bacteria, giving a larger biomass (Sieracki et al., 2006; Lami et al., 2007). In order to transport bacterial “biomass” to a higher eutrophic level, cell size is an important consideration for grazing by protists. Cell volume was reported for AAnPB in the open ocean (Sieracki et al., 2006; Lami et al., 2007); however, cell size, abundance and biomass data for AAnPB have not been reported for eutrophic aquaculture areas.

The phylogeny of AAnPB reported for culturable species indicates that AAnPB belong to *alpha-, beta-*, and *gamma-proteobacterial* (Yurkov and Csotonyi, 2009) and *Gemmatimonadetes* clades (Zeng et al., 2014). Uncultured groups are known to be phylogenetically different from the culturable ones (Yutin et al., 2007; Ferrera et al., 2014). The phylogenetic composition was variable among environments (Yutin et al., 2007) and across seasons (Ferrera et al., 2014). The role of bacteria in coastal sea areas is important to the understanding the matter cycling in eutrophic areas with aquaculture where excess organic matter is supplied. Although the AAnPB in the aquaculture sites have not been studied to date, our previous report (Sato-Takabe et al., 2015) proposes a hypothesis of high abundance in eutrophic aquaculture sites due to the dual energy production ability.

The Uwa Sea in the southwestern part of the Seto Inland Sea in Japan has been the site of the most productive aquaculture area in Japan for many years. This area has been used for concentrated fish and pearl oyster aquaculture for more than 50 years. One of the important factors for sustaining aquaculture in this area is the unique ocean structure that can deliver warm surface water and nutrient-rich upwell water from the Pacific Ocean (Takeoka and Yoshimura, 1988; Kaneda et al., 2002). Another factor is expected to be matter cycling from the microbial loop to the classical food web. The aim of the present paper is to determine the temporal abundance and species diversity of AAnPB in the Uwa Sea, and assess the effectiveness of this compartment in the transport of organic matter to higher trophic levels in the aquaculture area.

## Materials and Methods

### Study Site and Sampling

**FIGURE 1:**
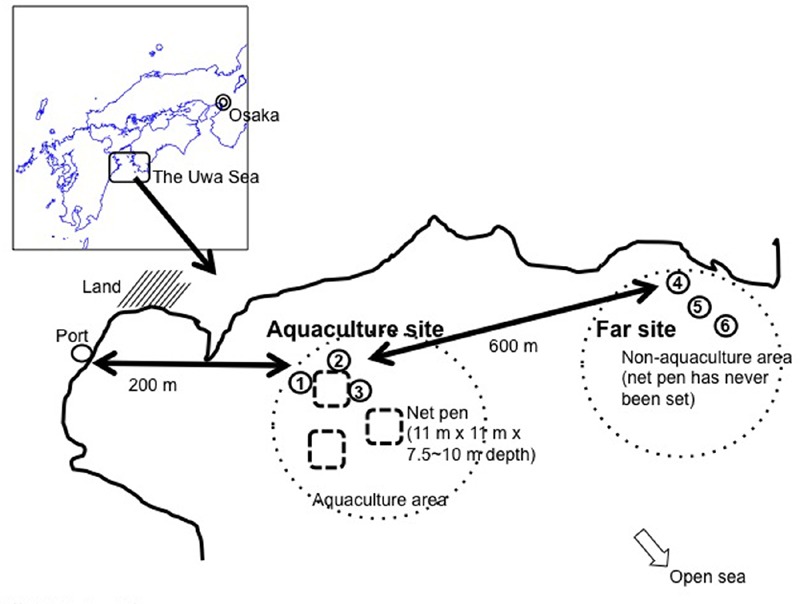
**Map of sampling sites.** Net pen size and distances are indicated.

Sampling sites were in the red seabream (*Pagrus major*) aquaculture area, which is located in the Uwa Sea, a coastal area of Shikoku Island in southwestern Japan (**Figure 1**). The water depth in the area is 30–50 m. Water sampling was conducted at three sites near the net pen (aquaculture site ID; EH-1, EH-2, and EH-3) on October 24 and December 26, 2012 and March 5, April 19, May 15, June 4, July 2, September 18, and October 16, 2013. All samplings were performed at morning (9:00–11:00 am). The net pen has dimensions of 11 m × 11 m with a depth of 7.5–10 m and is used for red seabream rearing. The monthly and cumulative feeding amount given to one net pen from April to October is summarized in **Supplementary Figure S1**, indicating cumulative feeding amount was over 30,000 kg in this duration. This shows a high concentration of organic matter was administered, thus we defined this area as an eutrophic one. Sampling was also conducted at three sites about 600 m away from the aquaculture area (far site ID; EH-4, EH-5, and EH-6). Water at these six sites was collected with a Van-Dorn or Niskin sampling bottle at depths of 1 and 20 m. Concentration of dissolved oxygen (DO) ranged from 87 to 100% in these depths. In addition, the bottom of sampling sites around pen net is sandy with shell exoskeleton. Even if deep waters bring up the bottom materials, Anoxic-PB could not affect on the AAnPB cell number measurement.

Environmental parameters (water temperature and salinity) were measured using field equipment (pH/COND METER D-54, HORIBA, Japan; Hand-Held Refractometer ATC-S/Mill-E, ATAGO, Japan). Sample water was dispensed into acid-washed 500 or 1000 ml polycarbonate bottles that had been rinsed three times with small amounts of collected seawater. Water temperature in aquaculture sites from October 2012 to October 2013 ranged from 15.6 to 27.3°C, and the salinity ranged from 31.5 to 37.0. Environmental characteristics of each sampling site are shown in **Supplementary Table S1**.

### Epifluorescence Microscopy (EM) and Infrared Epifluorescence Microscopy (IREM)

**FIGURE 2:**
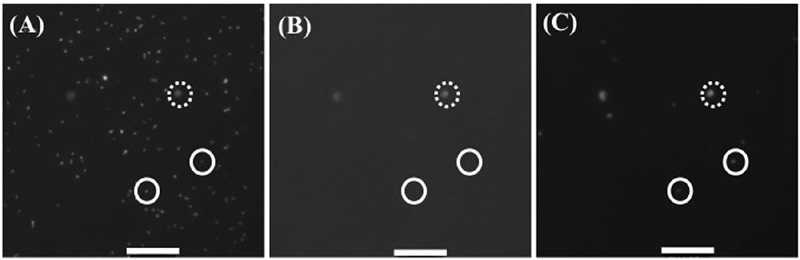
**Representative microscopic image of (A)** DAPI stain for total cells, **(B)** Chl a self-fluorescence (Cyanobacteria, autotrophic nano flagellate and eukaryotic phytoplankton) and **(C)** both Chl a positive (Cyanobacteria, autotrophic nano flagellate and eukaryotic phytoplankton) and BChl a positive (AAnPB) cells. Circle with solid line is AAnPB and with dotted line is other Chl a positive microbes. Scale bar indicates 100 pixel = 13.05 μm.

All samples (10 ml aliquots) were fixed with neutral formalin (1.0%, final concentration), incubated overnight at 4°C in the dark, and filtered through Nuclepore black polycarbonate membrane filters (0.2 μm in pore size) under gentle vacuum (≤20 cm Hg). The filters were dried and then stained with 4′,6-diamidino-2-phenylindole (DAPI) prepared at 1 μg ml^-1^ in a 3:1 mixture of Citifluor AF1 (Citifluor Ltd, London, United Kingdom) to Vectashield (Vector Labs, Burlingame, ON, Canada). Bacterial cells were enumerated on images taken on a Zeiss Axioplan 2 epifluorescence microscope (Carl Zeiss, Germany) equipped with a mercury lamp (USH-102D, Ushio, Japan) and a Photometrics CH-250 cooled, slow scan, infrared (IR)-sensitive CCD camera (iKon-M, ANDOR, Belfast, Northern Ireland) and connected to a Windows PC. Images were taken with scanning at 1,024 pixels × 1,024 pixels with resolution of 0.1305 μm per pixel. The following three epifluorescence filter and mirror sets were used: (1) BChl *a* (excitation short pass filter, 400–530 nm; emission long pass filter, >850 nm, RG850, Edmund Optics, Barrington, NJ, USA; short pass dichroic mirror, >650 nm, XF-2072, Omega Optical, Brattleboro, VT, USA); (2) Chl *a* (excitation, 546 ± 12 nm, emission long pass filter, >590 nm; short pass dichroic mirror, >580 nm, ZEISS Filter set 15, 488015 – 0000, Carl Zeiss, Oberkochen, Germany); (3) DAPI (excitation, 365 ± 12 nm, emission long pass filter, >397 nm; short pass dichroic mirror, <395 nm, ZEISS Filter set 01, 488001 – 0000, Carl Zeiss). First, all DAPI-stained bacteria were recorded with the DAPI filter set (100 ms exposure). Then, Chl *a* autofluorescence was recorded with the Chl *a* filter set (100 ms exposure) to identify Chl *a*-containing organisms. Finally, IR emission (>850 nm) images were captured with the BChl *a* filter set, showing both AAnPB and phytoplankton (10 s exposure). Generally, between 10 and 15 sets of images were acquired from each DAPI-stained filter. The acquired images were saved and semi-manually analyzed with the aid of MetaMorph Software (MetaMorph, Molecular Device, Sunnyvale, CA, USA) to distinguish between heterotrophic bacteria, *Synechococcus* and AAnPB, for each sample. The contrast and brightness of images were manipulated using the imaging software (Meta Morph) with the ‘Top Hat’ process to differentiate the cell image from the background. AAnPB were identified as cells having DAPI and IR fluorescence but not Chl *a* fluorescence (**Figure 2**). The cell abundance and cell size for total bacteria (total count of 350∼1550 cells sample^-1^) and AAnPB (total count of 20–169 cells sample^-1^) were calculated from DAPI stain image, because both cells can be estimated with the same condition. The mean values of all of the measured cells were taken. Cell length was measured on the DAPI-stained image as the length of the long axis. Since the cell in the present data set were almost spherical or short rods, cell volume was calculated as a spherical shape using one-half of the cell length, *r*, and the following equation: *V* = 4/3 × π ×*r*^3^. For calculation of cell volume as a rod, the calculation value is estimated to be 3.3 times less than that calculated as a sphere (Zeder et al., 2011). Although these methods can detect Anoxic-PB also, as mentioned above, our sampling sites and depth do not contain bottom materials brought up from bottom, and microscopic observation did not show large particles where Anoxic-PB would present. Cell number and biomass should be results of AAnPB.

### Denaturing Gradient Gel Electrophoresis (DGGE) for 16S rRNA and *pufM* Genes

#### DNA Extraction

Seawater samples (250 ml) were filtrated through 0.2 μm Nucleopore filters (ADVANTEC, Tokyo, Japan) to trap bacterial cells. DNA was extracted from the filter using a CTAB extraction protocol (Dempster et al., 1999).

#### PCR Amplification

The bacterial 16S rRNA gene was amplified for 30 cycles in 50 μl mixtures containing 2.5 mM MgCl_2_, 0.2 mM dNTPs, 0.25 μM of each primer, EUBf341-GC-clamp and r534 (Muyzer et al., 1993), 16–423 ng of template DNA, and 1.25 U of Ex Taq polymerase (TaKaRa, Otsu, Japan). Amplification cycles included an initial denaturation step at 95°C for 1 min followed by 10 cycles of denaturation at 95°C for 45 s, annealing at 65 to 56°C (45 s, with decrease of 1°C each cycle from 65 to 56°C), extension at 72°C for 1 min, and was followed by 20 cycles of denaturation at 95°C for 45 s, annealing at 55°C for 45 s, extension at 72°C for 1 min and a final extension at 72°C for 10 min. The *pufM* gene, which encodes the M-subunit of the photosynthetic reaction center, was amplified by 2-step PCR, and further analysis was performed according to a previous report (Yutin et al., 2008). The primer sets used in the present study are pufM_uniF (5′-GGNAAYYTNTWYTAYAAYCCNTTYCA-3′) and pufM_WAW (5′-AYNGCRAACCACCANGCCCA-3′) for the first-step PCR, and pufM_uniF (5′-GGNAAYYTNTWYTAYAAYCCNTTYCA-3′) and GC_WAW (5′-CCGCCGCGCGGCGGGCGGGGCGGGGGCACGGGGAYNGCRAACCACCANGCCCA-3′) for the nested-PCR (Yutin et al., 2008). The 275 bp-long *pufM*-fragments generated by the nested PCR were used for DGGE analysis. Yutin et al. (2008) confirmed that there is no bias by the nested PCR based on a comparison between one-step and two-step PCR products. Aliquots (2 μl) of the PCR products for 16S rRNA and *pufM* genes were checked by electrophoresis on 1.5% (wt/vol) agarose gels stained with Gel Red (Biotium, San Francisco, CA, USA).

#### DGGE Analysis

Denaturing gradient gel electrophoresis was performed with a Bio-Rad DCode system with denaturing gradients containing 30–65% denaturants for the 16S rRNA gene and 30–70% denaturants for the *pufM* gene, where the 100% denaturants corresponded to 7 M urea and 40% (v/v) formamide. The concentration of acrylamide was 8% (w/v), and the acrylamide:bis-acrylamide ratio was 37.5:1. Aliquots of PCR product of 20 μl (16S rRNA gene) and 30 μl (*pufM*) were applied to the gel. The gels were run at 60 V in a 0.5 × TAE buffer (pH 8.0) at 60°C for 16 h for the 16S rRNA gene and in a 1 × TAE buffer at 63°C for 16 h for the *pufM* gene, and gels were stained with 50 μl of 500 ml^-1^ SYBR Gold (Invitrogen, Waltham, MA, USA). Digital images were obtained under UV illumination using the Molecular Imager ChemiDoc^TM^ XRS (Bio-Rad, Hercules, CA, USA) system and Image Lab software (Bio-Rad).

#### Reamplification of DGGE Band

Denaturing gradient gel electrophoresis bands were excised from the gel, washed in 1 ml sterilized water, and then cut into sections of approximately 1 mm^3^ and incubated for >24 h at 4°C. Aliquots (2 μl) of the incubation were used as templates in the second-round PCR described above. The re-amplified products were run on a DGGE gel again to confirm the re-amplification product.

#### Sequencing and Phylogenetic Analysis

The excised DGGE bands were sequenced directly. Bidirectional sequencing was performed using Genetic Analyzer 3730xl (Applied Biosystems, Foster City, CA, USA) after preparing the sequencing reaction with a BigDye terminator sequencing kit (ver. 3.1, Applied Biosystems) using EUBf341-GC-clamp and r534 primers for the 16S rRNA gene and pufM_uniF and GC_WAW primers for the *pufM* gene. In order to identify the phylogenetic position of the obtained sequences, closely related sequences were identified using BLAST (Altschul et al., 1997) targeted to the nr database in the DNA Data Bank of JAPAN (DDBJ^1^). Phylogenetic relationships from multiple sequences were inferred using the ARB software package (Ludwig et al., 2004). The obtained sequences and the closely related sequences were aligned to a non-redundant alignment of small subunit rRNA genes (SSU Ref_NR_99 ver. 119, SILVA database^2^) for the 16S rRNA gene using the SILVA incremental aligner (SINA) alignment service^3^. The alignment was corrected manually where correction was necessary. The aligned sequences were added to the reference tree, which was constructed from sequences in the Ref_NR alignment using the neighbor-joining (NJ) method with 2000 times bootstrap resampling without allowing a change in the overall topology on the basis of ARB Parsimony. A NJ tree for the *pufM* gene was constructed with nucleotide sequences of the partial *pufM* gene. The Jaccard coefficient was applied to calculate the similarity between DGGE profiles and between-group average linkages were used for clustering (PRIMER6; PRIMER-E Ltd, Auckland, New Zealand).

## Results and Discussion

### Abundance and Cell Volume of AAnPB

**FIGURE 3:**
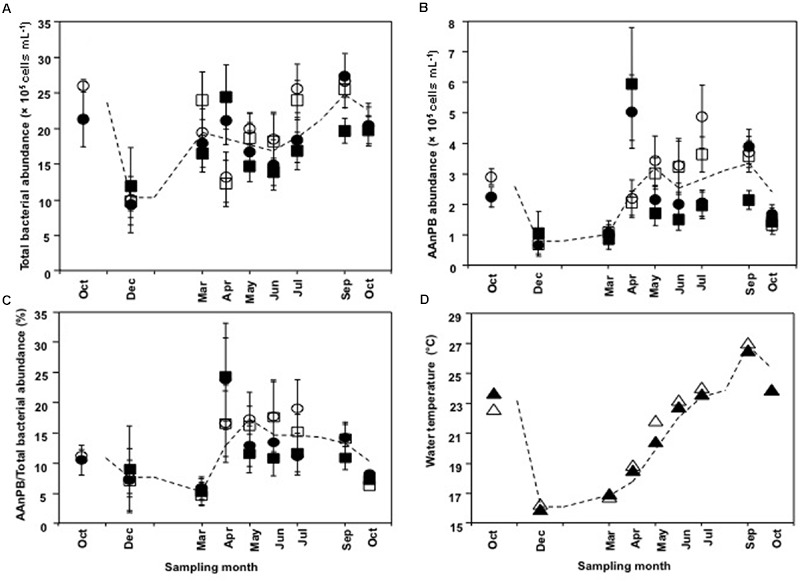
**Time course changes in (A)** total bacterial abundance, **(B)** AAnPB abundance, **(C)** AAnPB%, and **(D)** water temperature. Each value is average of three sites. Circles represent aquaculture sites and squares represent far sites. Water temperature is indicated by triangles. Sampling depths are 1 m (open symbols) and 20 m (closed symbols). Each symbol is the mean value, and the vertical bars indicate SD. The dashed lines indicate the simple average based on all plot data at each sampling site.

Abundances of total bacteria and AAnPB were calculated as the mean of the three aquaculture sites (EH-1, EH-2, and EH-3) and far sites (EH-4, EH-5, and EH-6). Change in bacterial abundance through the year is shown in **Figure 3**. Total bacterial number ranged from 9.4 × 10^5^ to 2.7 × 10^6^ cells ml^-1^ (**Figure 3A**) and that of AAnPB ranged from 6.8 × 10^4^ to 5.9 × 10^5^ cells ml^-1^ (**Figure 3B**). This indicates that AAnPB accounted for 4.7–24% of total bacteria (**Figure 3C**). Increasing AAnPB abundance from March occurred coincidently with raising water temperature (**Figures 3B,D**), whereas total bacteria abundance was not correlated with water temperature. AAnPB abundance is reported to respond to temperature, although the correlation is not strong (Mašín et al., 2006; Zhang and Jiao, 2007). The present result might be caused by difference of temperature response of total bacteria and AAnPB. Cell abundance in May–July (summer season) at the aquaculture sites was not statistically significantly different from the cell abundances of total bacteria (*P* = 0.537, two-tailed *t*-test) and AAnPB (*P* = 0.417, Mann–Whitney Rank Sum Test, *U* test). Differences with depth (1 and 20 m) between May and July were observed, suggesting an abundance of cells at the surface during the stratified period. Zhang and Jiao (2007) mentioned that high abundance patches of heterotrophic bacteria, including AAnPB, were supported by dissolved organic carbon (DOC) rather than temperature. In previous studies from various ocean environments, the percentage of AAnPB was 0.1–24% (Schwalbach and Furhman, 2005; Lami et al., 2007). The highest abundance of 24% of total bacteria was observed in an oligotrophic area of the South Pacific Ocean (Lami et al., 2007). AAnPB are suggested to have supplementary energy production utilizing light when low organic matter conditions are encountered. Although AAnPB were detected in epicontinental seas (Zhang and Jiao, 2007; Chen et al., 2011) and inland seas (Mašín et al., 2006), abundance under such high organic matter conditions was much lower than in oligotrophic seas (Mašín et al., 2006; Zhang and Jiao, 2007; Chen et al., 2011). Observations in our present study showed that the percentage of AAnPB was 4.7–24%, which was the highest among reports available to date (Mašín et al., 2006; Zhang and Jiao, 2007; Chen et al., 2011). Our previous report showed 3.5–7.9% in open areas of the Uwa Sea (Sato-Takabe et al., 2015). This indicates that AAnPB contribute greatly to the bacterial carbon stock in the high organic matter concentration area of the Uwa Sea. Abundance, growth, cell volume and species diversity are factors that determine the contribution of bacteria in the ecosystem.

Previous studies reported that AAnPB grow faster than heterotrophic bacteria in the euphotic zone across the Atlantic Ocean (Koblížek et al., 2007) and consumed leucine with a high rate in the bacterial community in the Delaware Estuary (Stegman et al., 2014). Based on the results of our study and previous studies taken together, it is suggested that AAnPB can utilize organic matter more efficiently than other bacteria, which leads to fast growth and results in high abundance.

To estimate cell volume, we measured cell length. The cell length for total bacteria was 1.00 to 1.58 μm (median value, 1.280 μm; 25th percentile value, 1.207 and 75th percentile value, 1.370; *n* = 75) and that for AAnPB bacteria was 1.26 to 2.37 μm (median value, 1.647; 25th percentile value, 1.518; and 75th percentile value, 1.749; *n* = 75), showing that AAnPB were significantly larger than other bacteria (*P* < 0.001, Mann–Whitney rank sum test). Average cell volume of total bacteria was 1.17 ± 0.356 μm^3^ (*n* = 75), whereas that of AAnPB was 2.58 ± 1.15 μm^3^ (*n* = 75). We previously demonstrated that AAnPB were larger among total bacteria from open areas of the Uwa Sea (Sato-Takabe et al., 2015). The results of the present study focused on an aquaculture site are consistent with findings from a non-aquaculture area of the Uwa Sea, where the biovolume of AAnPB was 9.7–22% (Sato-Takabe et al., 2015). The AAnPB biomass was estimated to make up 10–53% of the total bacteria, suggesting that AAnPB account for a large part of bacterial carbon stock in the aquaculture area. Organic matter input (**Supplementary Figure S1**) would make the cell size of AAnPB larger in the present study area. Former reports (Sieracki et al., 2006; Lami et al., 2007) obtained from oligotrophic open oceans showed one third to three order magnitude smaller biovolume than our result.

### Community Structure through the Year

**FIGURE 4:**
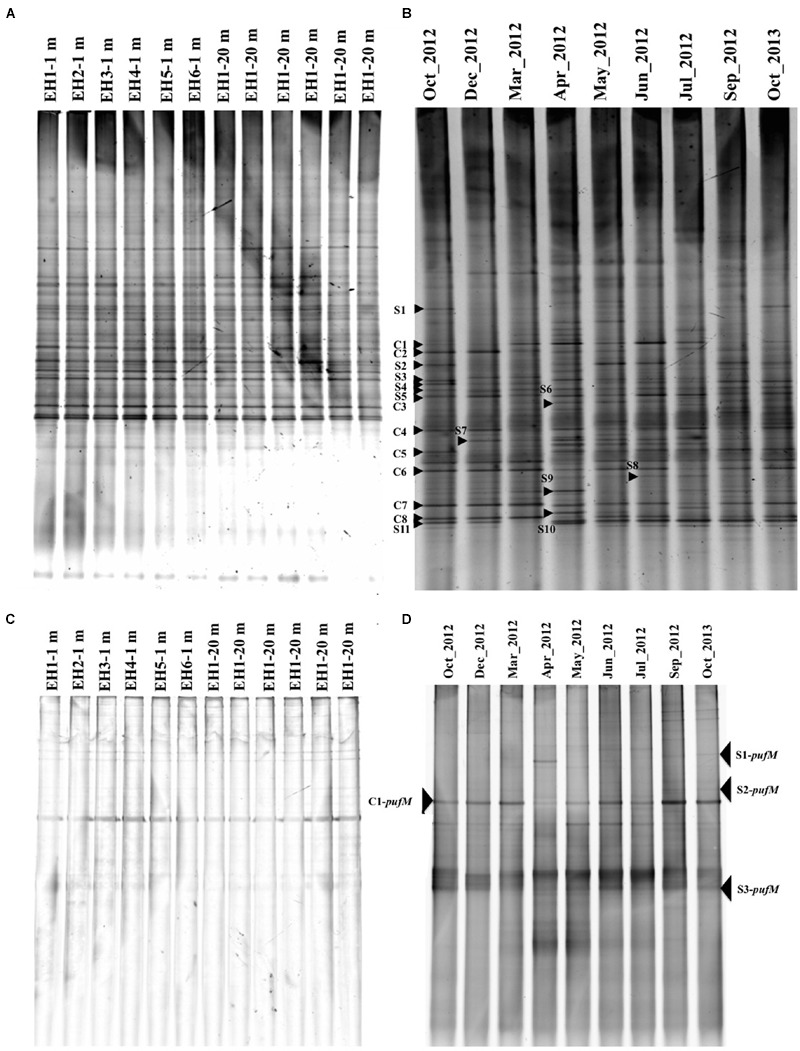
**Denaturing gradient gel electrophoresis (DGGE) images of 16S rRNA genes at six sites in October 2013 (A)** and time course changes at a depth of 1 m at site EH-1 from October 2012 to October 2013 **(B)**. DGGE images of the *pufM* gene in October 2013 **(C)** and time course changes at a depth of 1 m at site EH-1 from October 2012 to October 2013 **(D)**. Common bands (C series) and specific bands (S series) from the months indicated with an arrowhead and number were sequenced. The similarity of the banding profile is shown in **Supplementary Figure S2** as a tree constructed using the between-group average linkage method for clusters with the PRIMER 6 software.

The temporal and spatial changes in bacterial community composition were monitored by DGGE targeting the 16S rRNA gene, whereas changes in AAnPB were monitored with the *pufM* gene. All DGGE gel profiles are shown in **Figure 4** and the similarity is shown in **Supplementary Figure S2**. For the total bacterial community (16S rRNA gene), the profiles at depths of 1 and 20 m at all sampling sites (EH-1 ∼ 6) were similar (**Figure 4A**) and the similarity was >90% (**Supplementary Figure S2A**). This suggests that the bacterial community in this area is rather homogeneous in both depths of 1 and 20 m. Since high similarity was found among sampling sites throughout the year, representative data for year-long samples at a site (EH-1 at 1 m depth) were shown as **Figure 4B**. Among the bands in the DGGE gel, the season-common bands (C series bands) and season-specific bands (S series bands) were detected. Non-aquaculture sites are 600 m away from the aquaculture sites and no inlet of river and wastewater. However, residue of fish feed might draft to the non-aquaculture sites, which probably gave similar nutrient condition and bacterial flora.

In the case of AAnPB diversity, all sites showed quite similar profiles also (**Figure 4C**), suggesting that the same AAnPB are uniformly abundant in this area. Temporal changes in the *pufM* profile, however, were found as shown in **Figure 4D** (site EH-1, 1 m depth). Ferrera et al. (2014) reported seasonal succession in the Mediterranean Sea, suggesting the distribution of different subpopulations over time. Our results also indicate that AAnPB are temporally highly dynamic. The phylogenetic relationship of the season-common bands (C-*pufM* series) and season-specific bands (S-*pufM* series) are discussed below.

### Phylogenetic Diversity of Total Bacteria

**FIGURE 5:**
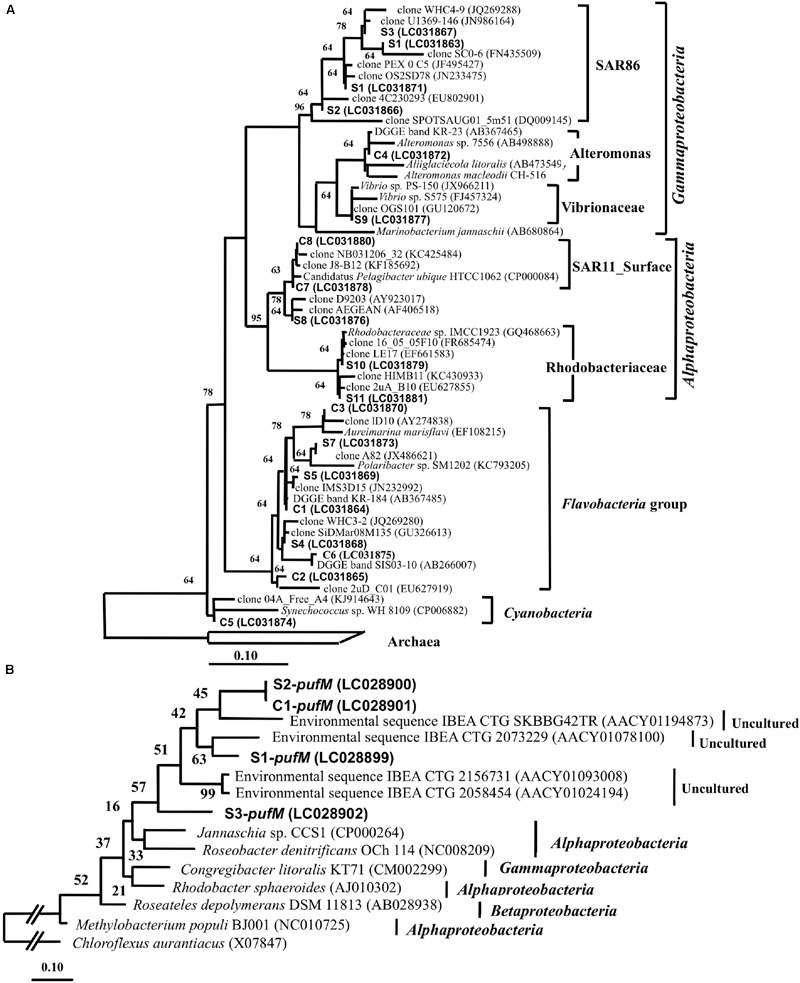
**(A)** A neighbor-joining tree of 16S rRNA gene sequence (155 bp) excised from the DGGE gel. C series are all season-common bands, and S series are season-specific bands. The scale bar represents 10% estimated sequence divergence. Short DGGE band sequences were added by ARB_PARSIMONY. **(B)** Neighbor-joining tree of *pufM* gene sequences (138 bp) excised from DGGE gel. The all season-common band is designated as *pufM*-C and the season-specific band as *pufM*-S. The scale bar represents 10% estimated sequence divergence. The numbers next to each branch indicate confidence values.

Denaturing gradient gel electrophoresis bands of 16S rRNA gene were excised from the gel in order to identify the major bacteria at the EH-1 site (1 m depth) (**Figure 5A**). Season-common bands included *Flavobacteria* (band# C1, 2, 3, and 6), *Gammaproteobacteria* (C4), *Alphaproteobacteria* (C7 and 8) and cyanobacteria (C5). Season-specific bands were identified as *Flavobacteria* (S4, S5, and S7), *Gammaproteobacteria* (S1, S2, S3, S6, and S9), and *Alphaproteobacteria* (S8, S10, and S11). These dominant phylogenetic groups are in accordance with other reports from coastal seawater areas without aquaculture site (Yokokawa and Nagata, 2010), and aquaculture facilities (Martins et al., 2013). The season-common bands (*Flavobacteria* and *Gammaproteobacteria*) are known to be active growers (Schäfer et al., 2001; Taniguchi and Hamasaki, 2008; Ferrera et al., 2011), suggesting their role in organic matter transport to the upper trophic level by grazing. DGGE sensitivity was reported by Muyzer et al. (1993) and Murray et al. (1996), who showed that 1–2% of bacterial populations in the mixed assemblage of selected bacterial strains appeared as bands. The bands showing high abundance in the present study are estimated to represent cell density of 10^4^∼10^5^ cells ml^-1^. These bands suggest that this area has sufficient abundance of major heterotrophic bacteria, which represent key groups in organic matter cycling in the microbial loop in this ecosystem.

### Phylogenetic Diversity of AAnPB

Partial *pufM* sequences were determined for four bands excised from the DGGE gel for the EH-1 site (1 m depth) sample. The season-common band (C1-*pufM*) was detected at all sites (**Figure 4C**), and the season-specific bands (S1 ∼ S3-*puf*M) were also detected. The sequences of these bands showed that all were closely related to uncultured IBEA_CTG clones (**Figure 5B**). The sequence identities to IBEA_CGT SKBBG42TR clone were as follows: C1-pufM 75%, S2-pufM 76%, S1-pufM 79%, and S3-pufM 72%. Similarity to other clones was lower than SKBBG42TR case (Yutin et al., 2007, Yutin et al., 2008). Yutin et al. (2007) reported that the phylogroups including IBEA_CTG clones were the main members of AAnPB found in oligotrophic regions. The present results suggest that the phylogroup closely related to IBEA_CTG clones would be abundant in not only the open ocean but also coastal eutrophic sea areas. These bacteria were the first described phylogroups based on analysis of the *puf*M sequence. The present study indicates that the new AAnPB phylogroups are abundant in the Uwa Sea, which should play a role to transport of organic matter to higher eutrophic levels.

In summary, the present study shows that AAnPB contribute 10–53% of the bacterial biomass and account for a large part of the bacterial carbon stock around eutrophic aquaculture areas. The abundant phylogroups might be ubiquitously abundant in the oligotrophic open ocean and eutrophic coastal sea areas. These AAnPB groups are possibly some of the key bacteria for organic matter transfer to protists as well as major proteobacteria.

## Author Contributions

YS-T: experiment and writing draft. HN and TK: experiment. TY and KO: sampling. KH: microscope. SS: planning and writing manuscript.

## Conflict of Interest Statement

The authors declare that the research was conducted in the absence of any commercial or financial relationships that could be construed as a potential conflict of interest.

## Footnotes

1 http://www.ddbj.nig.ac.jp/

2 http://www.arb-silva.de

3 http://www.arb-silva.de/aligner/

## Supplementary Material

FIGURE S1 **Feed amount to net pen with three sampling sites (EH-1, -2, and -3).** Closed bar is cumulative food amount and open bar is monthly administration amount. Feed is Moist-Pellet, which is made from fish-meal and mixed with water immediately before administration to get the pellet soft.

FIGURE S2 **The similarity of the banding profile shown in **Figure 4** as a tree constructed using the between-group average linkage method for clusters with the PRIMER 6 software**.

TABLE S1 **Environmental characteristics of sampling sites around the aquaculture site in the Uwa Sea, Japan**.
