# Supplementary figures and images for: Abundance of Common Aerobic Anoxygenic Phototrophic Bacteria in a Coastal Aquaculture Area
Source: Front Microbiol. 2016 Dec 15;7:1996. doi: 10.3389/fmicb.2016.01996 (PMC5156720; doi:10.3389/fmicb.2016.01996)

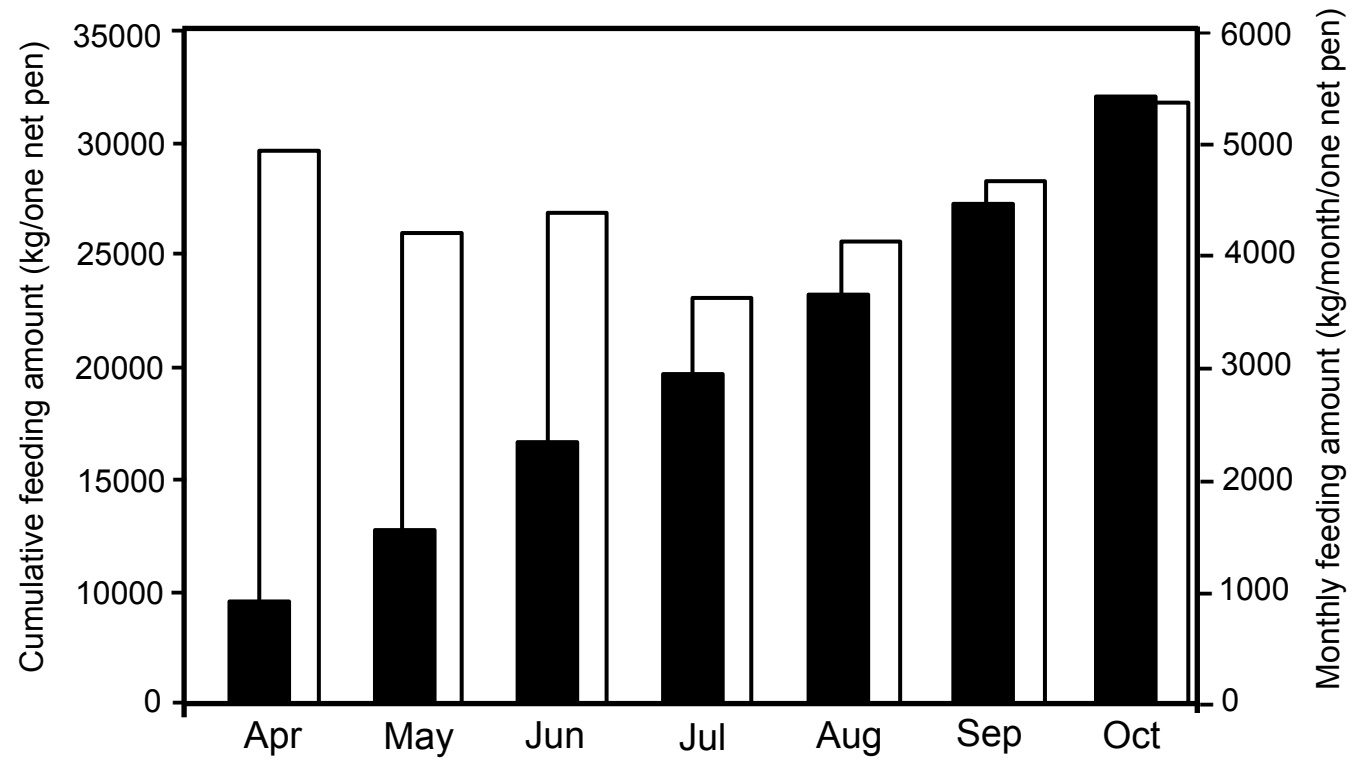

Fig. S1 (Sato-Takabe et al)

Supplement: FIGURE S1 — Feed amount to net pen with three sampling sites (EH-1, -2, and -3). Closed bar is cumulative food amount and open bar is monthly administration amount. Feed is Moist-Pellet, which is made from fish-meal and mixed with water immediately before administration to get the pellet soft. [file Presentation_1.PDF]

**A**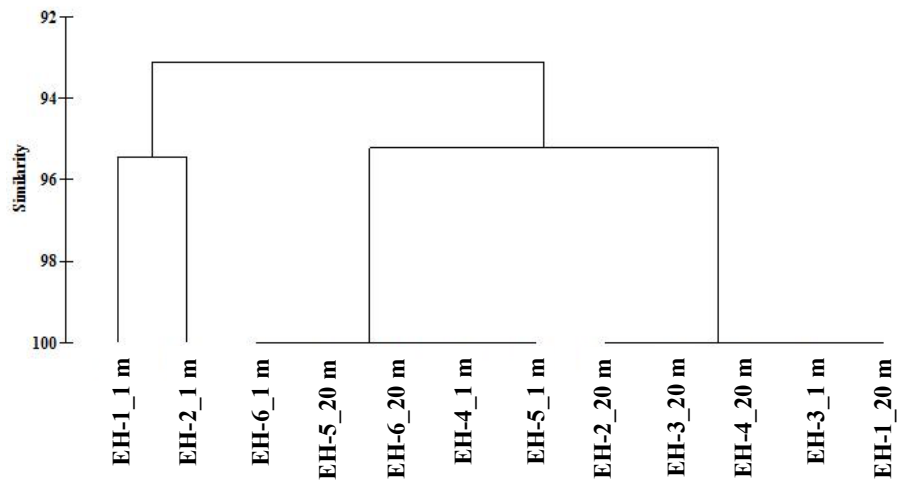**B**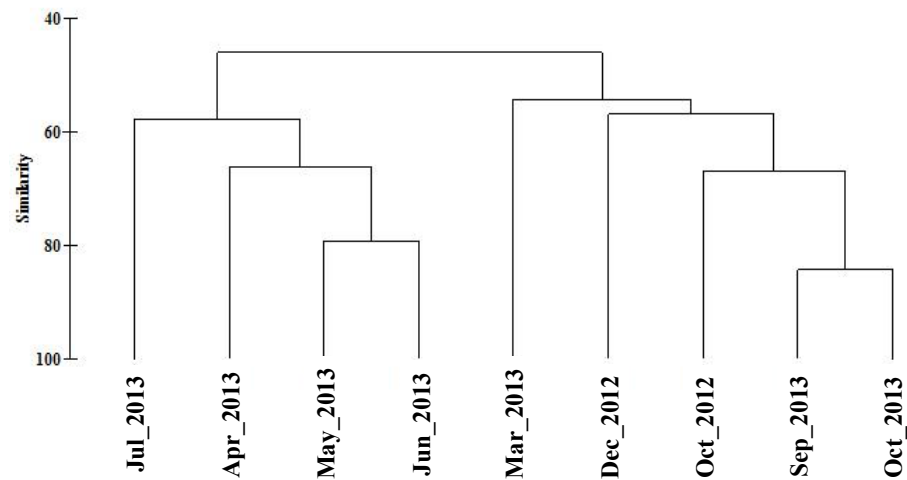**C**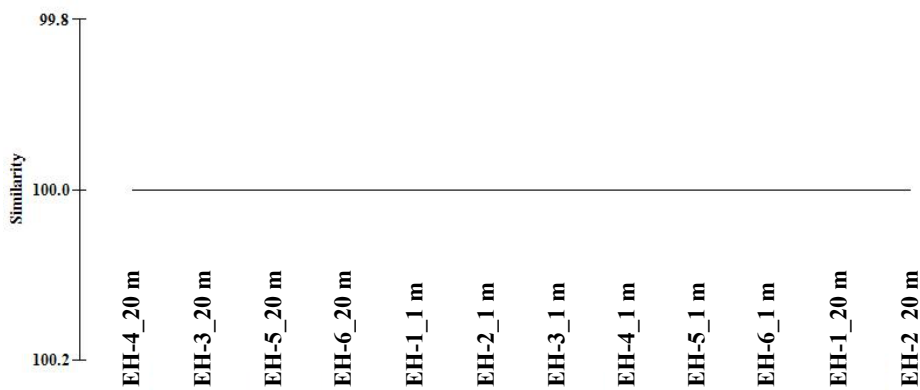**D**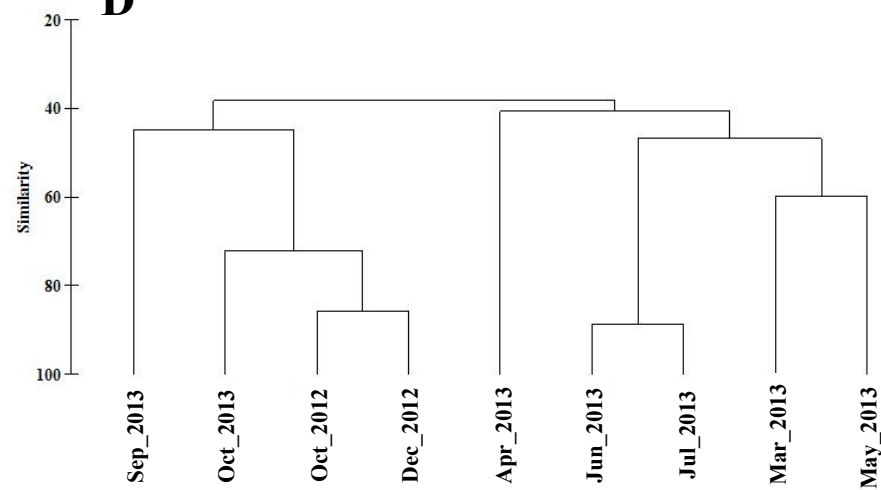

Fig. S2 (Sato-Takabe et al.)

Supplement: FIGURE S2 — The similarity of the banding profile shown in Figure 4 as a tree constructed using the between-group average linkage method for clusters with the PRIMER 6 software. [file Presentation_2.PDF]
