# Supplementary material for: Abundance of Common Aerobic Anoxygenic Phototrophic Bacteria in a Coastal Aquaculture Area
Source: Front Microbiol. 2016 Dec 15;7:1996. doi: 10.3389/fmicb.2016.01996 (PMC5156720; doi:10.3389/fmicb.2016.01996)
Supplement: TABLE S1 — Environmental characteristics of sampling sites around the aquaculture site in the Uwa Sea, Japan. [file Table_1.DOCX]

**Table S1** Environmental characteristics of sampling sites around the aquaculture site in the Uwa Sea, Japan.

**Table S1** continued
